# Bullous pemphigoid associated with squamous cell lung carcinoma showing remarkable response to carboplatin-based chemotherapy:  a case report

**DOI:** 10.1186/s13256-022-03323-9

**Published:** 2022-05-05

**Authors:** Prajwol Shrestha, Mathew K. George, Sweta Baidya, Sunil K. Rai

**Affiliations:** 1 Northwest Cancer Centre, Tamworth Hospital, Dean Street, Tamworth, NSW 2340 Australia; 2 Chris O’Brien Lifehouse, Missenden Road, Camperdown, NSW 2050 Australia; 3 Campbelltown Hospital, Therry Road, Campbelltown, NSW 2560 Australia

**Keywords:** Bullous pemphigoid, Squamous cell lung carcinoma, Carboplatin, Chemotherapy

## Abstract

**Background:**

Bullous pemphigoid is an uncommon dermatologic manifestation seen in squamous cell lung cancer, and evidence guiding optimal treatment, especially in the elderly population, is limited. We report herein a case of squamous cell lung cancer diagnosed after being investigated for refractory bullous pemphigoid showing marked response to carboplatin-based chemotherapy. This is the first case report that shows carboplatin can be used as an effective alternative in treatment of malignancy-associated bullous pemphigoid.

**Case report:**

An 80-year-old caucasian man developed extensive vesiculobullous rashes on his trunk, chest, abdomen, and inguinal region associated with significant pruritus causing sleep disturbance. The diagnosis of bullous pemphigoid was confirmed on skin biopsy. The skin lesions continued to worsen even after use of oral and topical steroid in addition to oral doxycycline. Chest computed tomography revealed a spiculated left lung lesion along with mediastinal lymphadenopathy. Fine-needle aspiration from the mediastinal lymph node confirmed metastatic squamous cell lung carcinoma. Carboplatin with gemcitabine was initiated, and significant response was seen within 3 days of chemotherapy. The skin lesions continued to remain in remission even after stopping the chemotherapy.

**Conclusion:**

Although there are still controversies regarding paraneoplastic etiology of bullous pemphigoid, this case presents a temporal association. It is the first case report showing a remarkable response with the use of a carboplatin-based regimen.

## Introduction

Several case reports have described the association of different types of malignancy with bullous pemphigoid, but a direct paraneoplastic correlation has not been proven. Bullous pemphigoid (BP) is a chronic, autoimmune, subepidermal, blistering skin disease mainly seen in elderly individuals.

Paraneoplastic or malignancy-associated BP differs from typical pemphigus, which is characterized by mucosal involvement and positive Nikolsky sign [2]. Paraneoplastic bullous pemphigoid is most commonly associated with B-cell lymphoproliferative disorders [3]. Bullous pemphigoid can also be associated with diabetes, rheumatoid arthritis, systemic lupus erythematosus, ulcerative colitis, multiple sclerosis, and myasthenia gravis and can be drug induced.^1^

Bullous pemphigoid is one of the rare paraneoplastic dermatologic manifestations in association with squamous cell lung cancer, and there is no clear evidence of response to carboplatin for its management.

## Case report

**Fig. 1:**
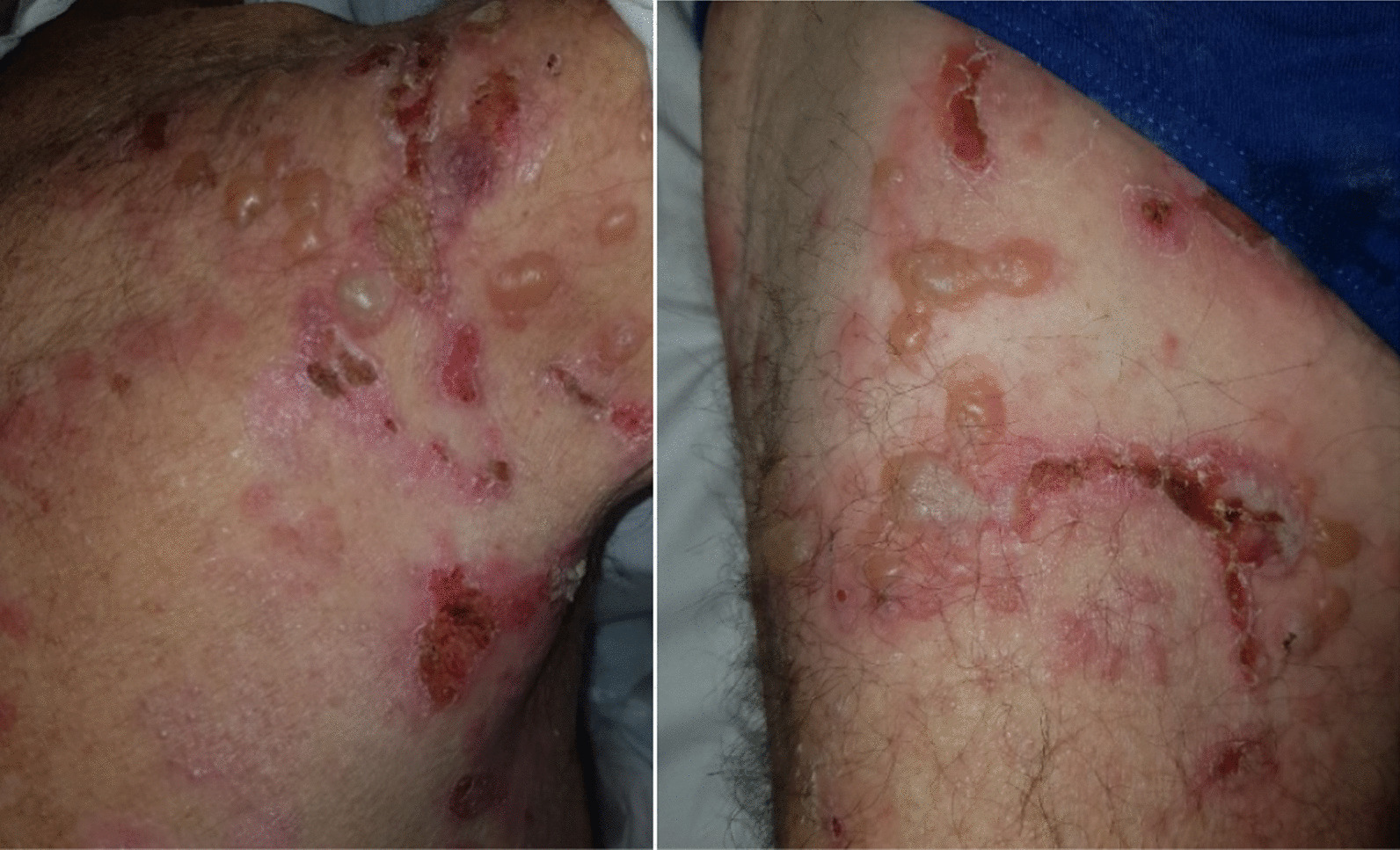
Pictures 1 and 2: day of admission (2 days before chemotherapy)

We report herein the case of an 80-year-old caucasian man who developed vesiculobullous rashes involving his trunk, shoulder close to the axilla, and inguinal regions for 2 months before admission on 17 June 2019 (Fig. 1, pictures 1 and 2). These were associated with severe pruritus causing significant sleep disturbance, and Nikolsky sign was negative. Clinical and histological findings were consistent with bullous pemphigoid (BP). His past medical history was only significant for chronic tophaceous gout and hypertension. He was a current smoker with 40 pack-years history of smoking. He lived alone and had a supportive friend who was the only close person in contact with him.

**Fig. 2:**
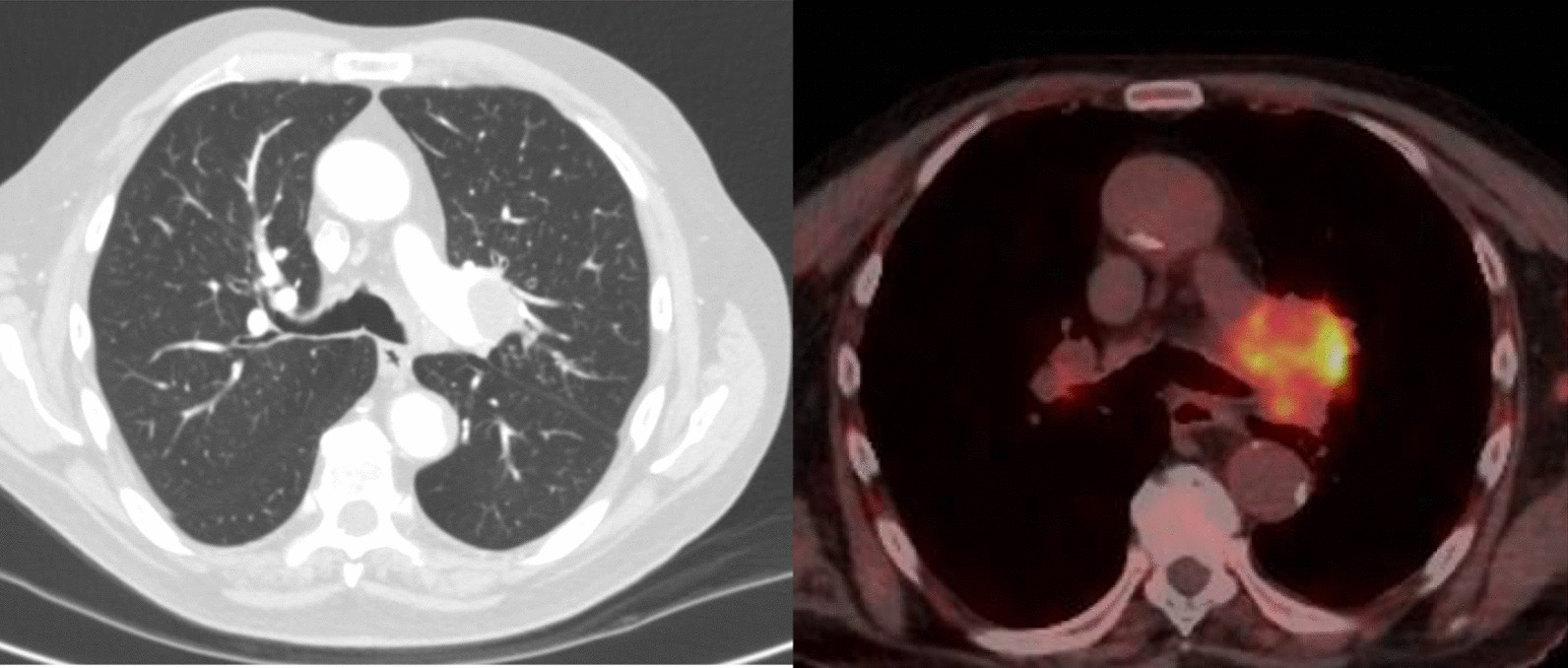
Chest CT showing left mediastinal lymphadenopathy (image A) with associated PET avid lung lesion (image B)

He was commenced on betamethasone cream and oral doxycycline followed by oral prednisone (1 mg/kg/day) daily without any response. Paraneoplastic nature of the disease was suspected, and computed tomography (CT) scan of the chest was performed given the significant history of smoking. CT scan (Fig. 2, image A) revealed a spiculated left lung lesion along with mediastinal lymphadenopathy. PET scan (Fig. 2, image B) showed a hypermetabolic focus on left lung apex mass suspicious of primary malignancy with metastases to the mediastinal nodes and liver lesion. Fine needle aspiration from the mediastinal lymph node confirmed advanced squamous cell lung cancer, which was PDL1 80%.

**Fig. 3:**
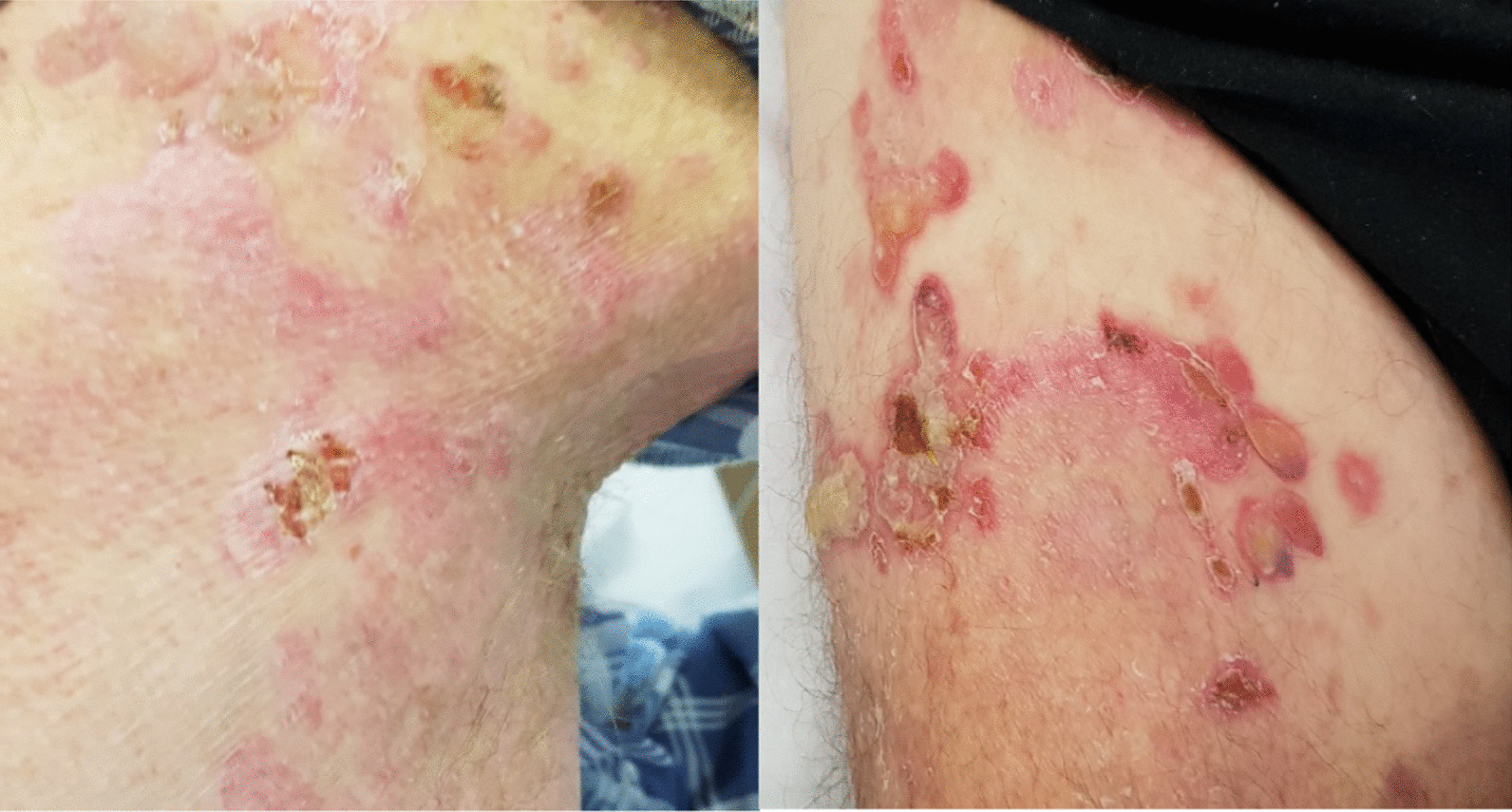
Pictures 3 and 4: day 3 post-chemotherapy

**Fig. 4:**
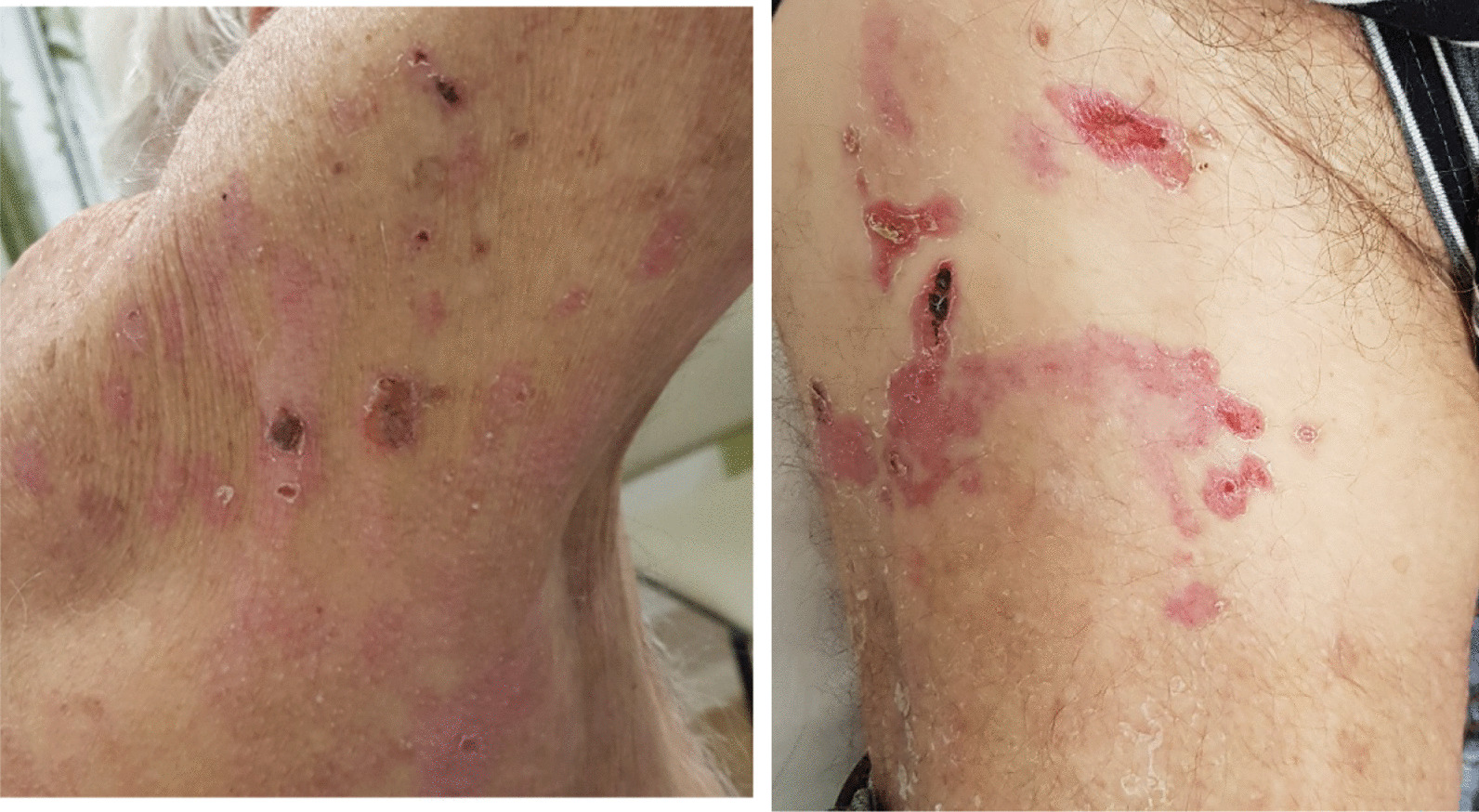
Pictures 5 and 6: day 14 of chemotherapy

**Fig. 5:**
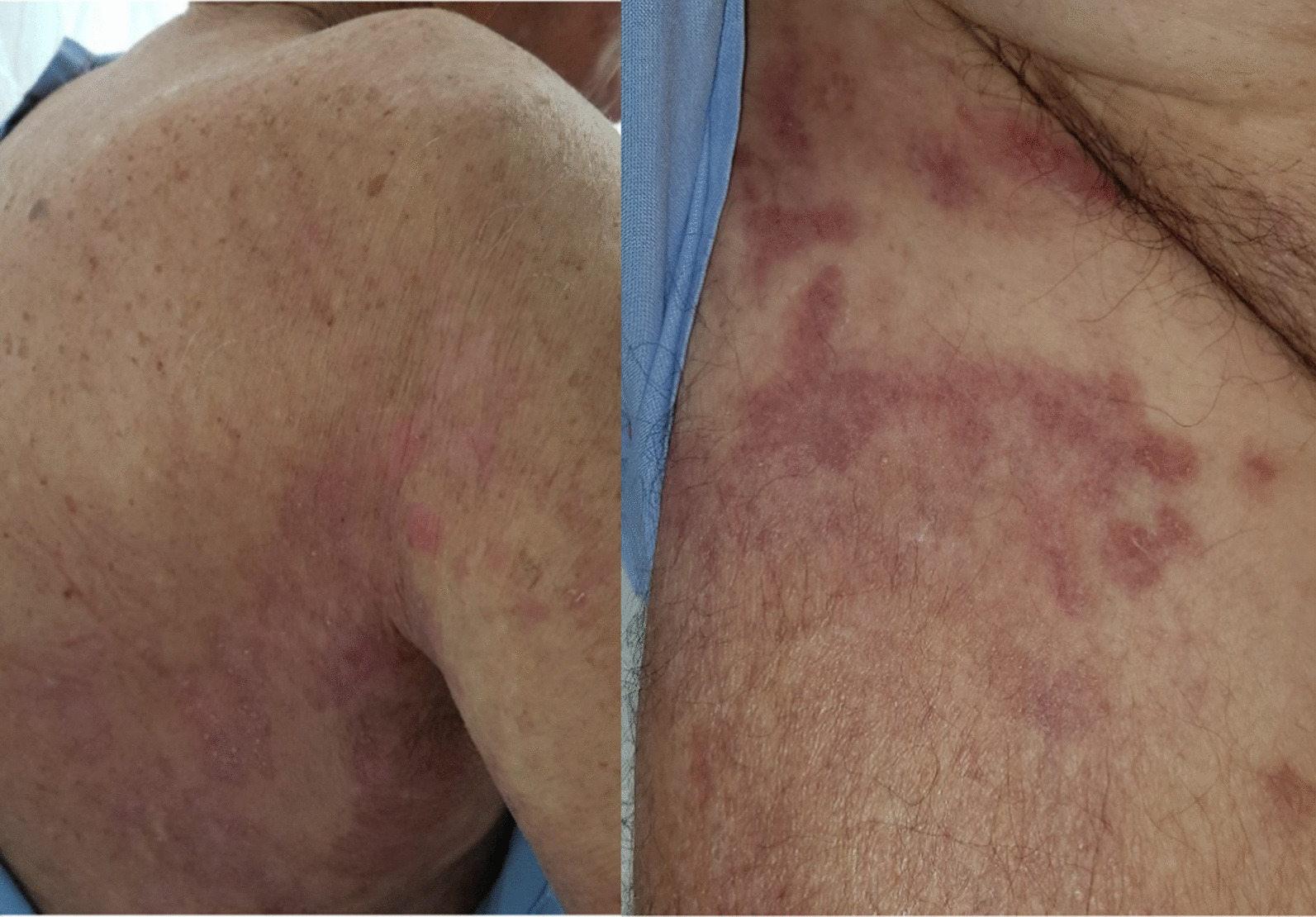
Pictures 7 and 8: week 5, chemotherapy stopped

Although eligible for first-line immunotherapy, because of the likelihood of autoimmune mechanism and association of bullous pemphigoid, the decision was made to treat with chemotherapy. He was treated with carboplatin and gemcitabine [23], based on a previous case report of BP associated with squamous cell carcinoma treated with cisplatin and gemcitabine. The dose was reduced at 75% of the calculated dose due to his age and high risk of toxicity predicted by Cancer and Aging Research Group (CAGR) chemotherapy toxicity calculator. A significant response was seen within 3 days of chemotherapy as depicted in Figs. 3 and 4 (pictures 3–6). But the subsequent dose of chemotherapy had to be withheld after he developed small bowel obstruction secondary to incarcerated inguinal hernia. Even after stopping chemotherapy, the bullous pemphigoid rashes continued to remain in remission after marked clinical improvement (Fig. 5, pictures 7 and 8). On further follow-up, he wanted to pursue best supportive care under palliative care team, hence further scans were not organized.

## Discussion and literature review

The paraneoplastic syndrome refers to metabolic or neuromuscular manifestations of certain malignancies, and these paraneoplastic manifestations are not attributable to direct tumor invasion or distant spread of tumor cells [4].

The paraneoplastic cutaneous syndrome is defined by two criteria: (i) the dermatosis must develop only after the development of cancer, and (ii) both dermatosis and cancer follow a parallel course in which complete removal of the cancer results in clearing of the dermatosis, while recurrence of cancer causes relapse of the dermatosis [5]. However, this definition is not without limitation, and exceptions such as pemphigus exist. Robinson et al. described rare forms of pemphigus, including paraneoplastic pemphigus in 1999 [2, 5]. Unlike paraneoplastic pemphigus, the paraneoplastic nature of bullous pemphigoid has not been accepted widely. Hence, some authors prefer to use the term pemphigoid associated with malignancies (PAM) [8].

Despite several published case reports and trials, a definite association remains controversial (Table 1). In their study involving over 1000 Japanese patients, Ogava et al. found a significantly higher incidence of malignancy in BP (5.8%) than that of controls aged over 70 years (0.6%) [13]. Similar associations are cited in Asia [14]. However, studies conducted in Sweden [15] and the USA [16] failed to prove any statistically significant association.

**Table 1:** Published case reports of BP associated with lung carcinoma and treatment response

S. no.	Publications reporting BP in lung cancer	Details	Treatment	Response
1	Rook et al. 1968 [17]	40 years with bronchial carcinoma	Surgery and high-dose corticosteroids	Partial response
2	Takeuchi et al. 1986 [6]	79-Year-old male, known lung cancer, diagnosed with BP Skin biopsy unknown	Radiotherapy	Lesions resolved during radiotherapy
3	Graham-Brown et al. 1987 [18]	74-Year-old male, bronchial carcinoma	Corticosteroid, azathioprine, surgery, and radiotherapy	Response only after surgery and radiotherapy
4	Sato et al. 1996 [7]	63-Year-old male, lung cancer BP confirmed on skin biopsy	Left upper lobectomy and mediastinal node dissection	Skin lesions resolved on postop. day 5
5	Shigemori et al. 2001 [19]	78-Year-old man noted to have undifferentiated large cell carcinoma on autopsy	–	–
6	Janah et al. 2014 [21]	52-Year-old Bronchial CA (squamous cell carcinoma)	Two cycles of cisplatin and Navelbine with topical steroid Chest and cerebellar RT	Good response
7	Das et al. 2015 [23]	76-year-old squamous cell CA of lung	Cisplatin and gemcitabine	Good response
8	Lakhdar et al. 2014	44-Year-old Small cell lung CA	Chemotherapy	Good response
9	Safini et al. 2015	46-year-old Small cell lung CA	Cisplatin and etoposide	Regression of 75% of cutaneous lesions

Since 1960, there have been case reports of bullous pemphigoid associated with lung carcinoma, although only a few include details of treatment in the metastatic setting. Data regarding the treatment regimen used for lung cancer in elderly patients with bullous pemphigoid are further limited. A systematic review from Balestri et al. in 2015 reported a total of 33 cases of solid tumors. The most common cancers were breast and colon cancer (*n* = 4).

Although Bullous pemphigoid associated with malignancies has been reported to be refractory to conventional treatment, most case reports have suggested a good response to cancer treatment, including surgery and chemotherapy [8, 17, 18, 21–23]. Rook et al. reported the first case of PAM in 1968 [17]. The patient was a 40-year-old with advanced bronchial carcinoma with partial response to high-dose corticosteroids and who remained in remission after surgery. In 1987, Graham-Brown et al. reported another case of bronchial carcinoma in a 74-year-old man who did not respond to corticosteroid and azathioprine. However, after surgery and radiotherapy, PAM was controlled with low-dose corticosteroid [18]. Shigemori et al. reported an autopsied case of a 78-year-old man with bullous pemphigoid who had undifferentiated large cell carcinoma of the lung in 2001 [19].

Another case of a 52-year-old chronic smoker with squamous cell lung carcinoma was reported in 2014 in the *Pan African Medical Journal*. The patient was treated with cisplatin and Navelbine, combined with chest and cerebellar radiotherapy along with local corticosteroid therapy, resulting in regression of bullous lesions after two courses of chemotherapy [21]. Das et al. also reported a case of a 76-year-old man with squamous cell carcinoma of the lung, which showed significant improvement after treatment with cisplatin and gemcitabine [23].

Two other case reports were published in Japanese in 1986 and 1996 [2, 6] but did not include details about histopathology. Bullous pemphigoid associated with small cell lung cancer was reported in a French publication by Lakhdar et al. in 2014. The patient was a 44-year-old smoker who had marked regression of the bullous lesions with chemotherapy [20]. Safini et al. also reported a case of bullous pemphigoid associated with small cell lung cancer in a 46-year-old man. He received cisplatin and etoposide, resulting in regression of 75% of cutaneous lesions after two cycles of chemotherapy [22].

We performed a literature search on Embase, Medline, and PubMed from 1996 to 18 August 2020. Table 1 summarizes the cases reported. Our observation and literature review suggest that bullous pemphigoid associated with malignancy may be underreported. The reported association with lung cancer is rare and even more so in case of squamous cell lung cancer. Almost all the reports suggest good response to various chemotherapy regimens that include cisplatin. However, to our knowledge, this is the first case report to show that carboplatin can be used as an effective alternative in treatment of malignancy-associated bullous pemphigoid. This is even more relevant as carboplatin is more commonly used in advanced lung cancer compared with cisplatin, more so in elderly population due to better tolerability.

## Conclusion

Many retrospective studies suggest increasing incidence of bullous pemphigoid [9–11], but large epidemiological studies have not justified extensive investigations and work-up to find underlying malignancy. We agree with the previously recommended approach of considering more extensive investigations in patients with history of malignancy, in younger age group, and cases with poor response to conventional immunosuppressants. While this case report presents a clear temporal association, and may be anecdotal experience, carboplatin-based chemotherapy in this setting can be considered as an effective alternative in palliative management of PAM.

## Data Availability

Not applicable, Additional details regarding the case are available for review in hospital medical records.

## Abbreviations

BP: Bullous pemphigoid
CAGR: Cancer and Aging Research Group
FNA: Fine needle aspiration
PAM: Pemphigoid associated with malignancies
PET: Positron emission tomography
